# Templated versus non-templated synthesis of benzo-21-crown-7 and the influence of substituents on its complexing properties

**DOI:** 10.3762/bjoc.6.14

**Published:** 2010-02-11

**Authors:** Wei Jiang, Christoph A Schalley

**Affiliations:** 1 Institut für Chemie und Biochemie, Freie Universität Berlin, Takustraße 3, 14195 Berlin, Germany

**Keywords:** benzo-21-crown-7, pseudorotaxane, self-sorting, supramolecular chemistry, template

## Abstract

Two procedures for the synthesis of benzo-21-crown-7 have been explored. The [1+1] macrocyclization with KBF_4_ as the template was found to be more efficient than the intramolecular macrocyclization without template. Pseudorotaxanes form with secondary ammonium ions bearing at least one alkyl chain narrow enough to slip into the crown ether. Substitution on benzo-21-crown-7 or on the secondary ammonium axle alters the binding affinity and binding mode. Compared to dibenzo-24-crown-8, the complexing properties of benzo-21-crown-7 turn out to be more susceptible to modifications at the crown periphery.

## Introduction

**Scheme 1:**
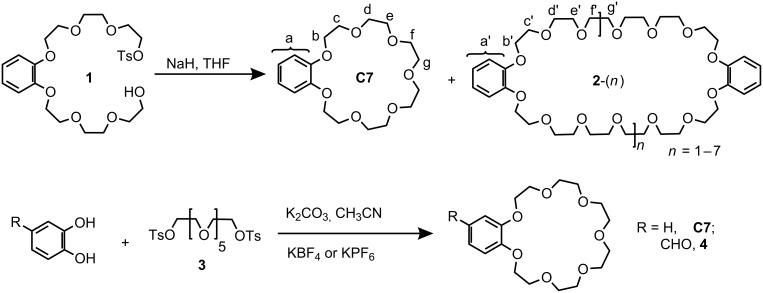
Two synthetic procedures for the preparation of benzo-21-crown-7 (**C7**) and its formyl analogue **4**: Top: The non-templated macrocyclization of **1** yields a mixture of crown ethers of different sizes. Bottom: With K^+^ as the template, benzo-21-crown-7 can be obtained in much better yields.

Mechanically interlocked structures [1–4] are attractive to chemists not only because they are aesthetically appealing but also due to their potential applications in molecular machines and smart materials [5–9]. Although a few covalent templates are known [10–12], their synthesis most often makes use of non-covalent templates [13–16], for which quite a number of different binding motifs are available that make the synthesis of many diverse and complex interlocked structures possible. Among these, the threaded interaction of secondary ammonium ions with larger crown ethers is a prominent example [17–22]. Recently, Huang and co-workers reported that the macrocycle size for forming pseudorotaxane can be reduced to only 21 atoms, namely benzo-21-crown-7 [23] (**C7**; Scheme 1) and pyrido-21-crown-7 [24], which could still slip over a secondary dialkylammonium ion when one of the alkyl groups is a narrow alkyl chain. By using this binding motif, the so far smallest [2]rotaxane consisting of only 76 atoms and having a molecular weight of not more than 510 Da was synthesized by Chiu and co-workers [25]. More recently, we applied **C7** together with dibenzo-24-crown-8 (DB24C8) to the construction of a four-component self-sorting system based on the fact that **C7** cannot pass over a phenyl stopper group at the end of a dialkylammonium axle, while DB24C8 can [26]. This system was further extended to construct more complex multiply interlocked structures by using the strategy of integrative self-sorting [26–27] which ensures programmability and positional control of all distinct subunits present in the complexes. Along this line, more diverse and complex supramolecular structures could be obtained when suitable instructions are written into the structures of their components.

Modification of crown ethers and their secondary ammonium guests allows variation of their binding properties and enables them to be incorporated into more complex assemblies [28]. In this respect, benzocrown ethers are more preferable than their aliphatic analogs due to the easy-to-achieve substitution on the benzene ring. One prerequisite for the generation of more complex supramolecular architecture based on such ammonium/crown binding motifs is the efficient synthesis of the building blocks. Here we report on attempts to improve the synthesis of **C7** and the preparation of substituted derivatives. Two synthetic routes, one which utilizes a templating cation and one which does not involve a template, are compared. Finally, the effects of substituents on the crown ether binding behavior are examined to lay the basis for a more precise control over the assembly of future complex assemblies.

## Results and Discussion

**Synthesis of C7.** Several synthetic procedures for **C7** have been explored systematically under phase-transfer conditions by Lukyanenko et al. [28]. Among them, intramolecular macrocyclization via monotosylate **1** generated in situ gives rise to the highest yield (68%). To test the efficiency of intramolecular ring closure in the absence of phase-transfer catalysis, we synthesized the monotosylate **1** which is then used in a separate macrocyclization (Scheme 1). Disappointingly, only 24% yield was achieved for the synthesis of **C7** from **1**. A second fraction of 31% turned out to be a mixture of **C7**’s bigger homologues **2**-(*n*) (*n* = 1−7) There are two reasons responsible for the relatively low yield: (i) the initial concentration (90 mM) of **1** is too high, favoring polycondensation over the intramolecular macrocyclization; (ii) the sodium ion originating from the NaH used as the base is not an appropriate template for **C7** [29]. Meanwhile, the low yield and long procedure discourage the application of intramolecular macrocylization to the synthesis of **C7**’s derivatives. Therefore, an alternative procedure with improved efficiency was sought.

The synthetic procedure with catechol and hexa(ethylene glycol) ditosylate (**3**) (Scheme 1) is advantageous since they are commercially available or easily prepared from commercially available materials. However, under phase-transfer conditions, this procedure gives **C7** in a relatively low yield (22%), which is not acceptable for synthesizing complex **C7** derivatives. Huang et al. [23] modified this procedure by introducing KPF_6_ as a template, which increased the yield to 69%. Nevertheless, we found it difficult to cleanly separate the KPF_6_ salt from **C7** during the reaction workup, since their complex dissolves well in organic solvents (e.g. CDCl_3_, ethylacetate). This can be attributed to the quite high hydrophobicity of the PF_6_^−^ anion. Instead, KBF_4_ was found to be a very good template which gives a satisfying yield (70%) and could be completely removed after column chromatography. This was further supported by the application to the synthesis of **4** (yield: 62%).

**Scheme 2:**
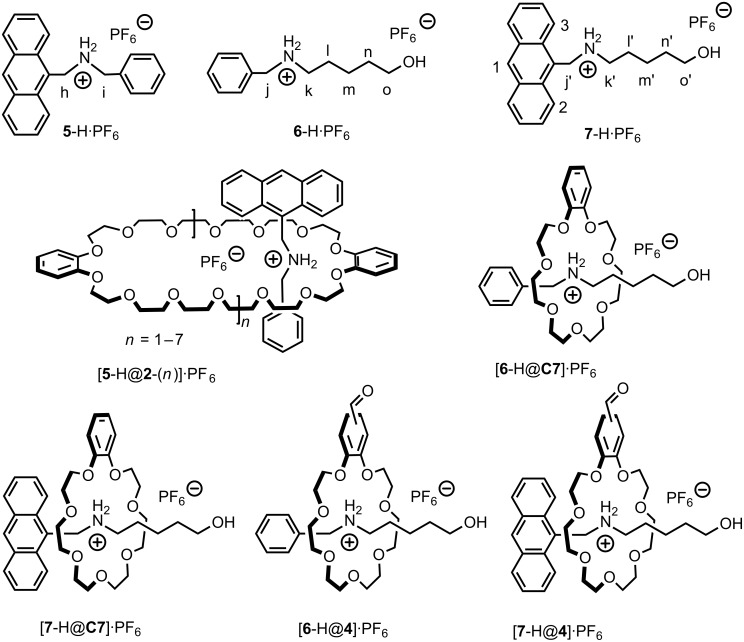
Molecular structures of guests **5**-H•PF_6_, **6**-H•PF_6_, and **7**-H•PF_6_, and their complexes with **2**-(*n*), **C7** and **4**.

**Figure 1:**
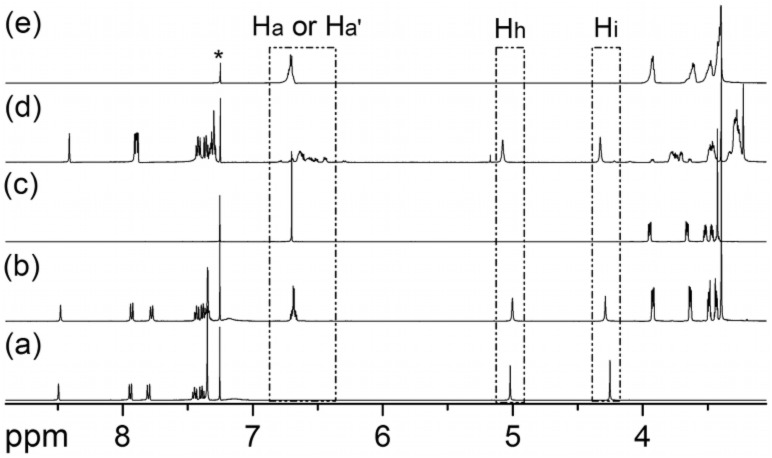
^1^H NMR spectra (500 MHz, 298 K, CDCl_3_:CD_3_CN = 2:1, 10.0 mM) of **5**-H•PF_6_ (**a**), mixture of **5**-H•PF_6_ and **C7** (**b**), **C7** (**c**), mixture of **5**-H•PF_6_ and **2**-(*n*) (**d**), and **2**-(*n*) (**e**). Asterisk = residual undeuterated solvent.

**Characterization of higher crown oligomers 2**-(***n***). The signals in the ^1^H NMR spectra of **2**-(*n*) (Figure 1e) appear at almost exactly the same position as those of **C7** (Figure 1c). The broadening of the signals is the only indication that the sample contains more than just **C7**. Consequently, it is difficult to distinguish the larger oligomers from **C7** by simple ^1^H NMR experiments. In the corresponding ESI mass spectra, the ionization efficiency is quite low. Some of the major components can be observed easily, but minor products are hard to detect. Therefore, we added charged guest **5**-H•PF_6_ (Scheme 2) to the mixture to (i) detect signal shifts in the NMR spectra characteristic for the formation of complexes and (ii) to facilitate the ionization of the crown ether oligomers as ammonium complexes. This guest will furthermore provide straightforward evidence for the formation of crown ethers larger than **C7**, because the phenyl group in **5**-H•PF_6_ is too bulky to thread through the cavity of **C7** [23]. Complex formation thus immediately indicates that the crown ether must have a larger cavity than **C7**. As seen in Figure 1b, the spectra of the equimolar mixture of **5**-H•PF_6_ and **C7** is the simple superimposition of their individual spectra (Figure 1a,Figure 1c). However, addition of **5**-H•PF_6_ to the fraction containing the larger oligomers **2**-(*n*) caused shifts of all signals for both of guest and host indicative of complex formation (Figure 1d,Figure 1e). From these experiments, we can conclude that crown ethers larger than **C7** have formed, but the composition of the fraction containing **2**-(*n*) is still not yet clear. From the structure of the starting material **1**, dibenzo-42-crown-14 (**2**-(1)) is certainly the most likely candidate, but even larger structures cannot be ruled out yet.

**Figure 2:**
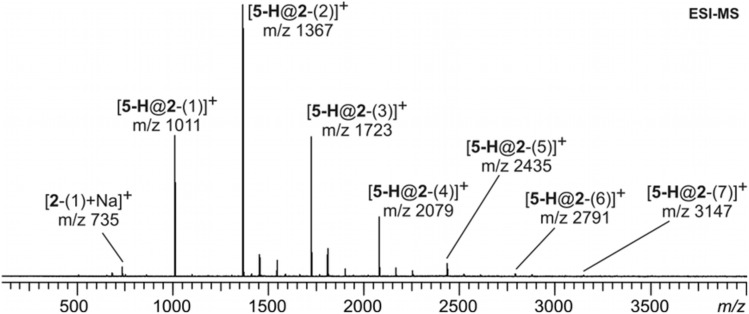
ESI-FTICR mass spectrum of a mixture of **5**-H•PF_6_ and **2**-(*n*) in dichloromethane.

To further elucidate the structure of **2**-(*n*), ESI-MS experiments were performed with the mixture of the second crown ether fraction and **5**-H•PF_6_. To our surprise, a broad series of several peaks evenly spaced by a distance of Δm = 356 amu was observed in the ESI mass spectrum (Figure 2). Considering that [**5**-H]^+^ does not simultaneously form complexes with several **C7** crown ethers, this peak distribution can only be assigned to a series of macrocycles with different sizes ranging from dibenzo-42-crown-14 (**2**-(1)) up to heptabenzo-168-crown-56 (**2**-(7)). Although the peak intensity does not necessarily reflect the solution composition quantitatively [30], the mass spectra indicate **2**-(1) − **2**-(4) to be the major components in the mixture, while the larger crown ethers are likely present only in trace amounts. Since we are focusing on **C7**, no attempt was made to separate the larger crown ethers by more sophisticated methods such as HPLC.

**Figure 3:**
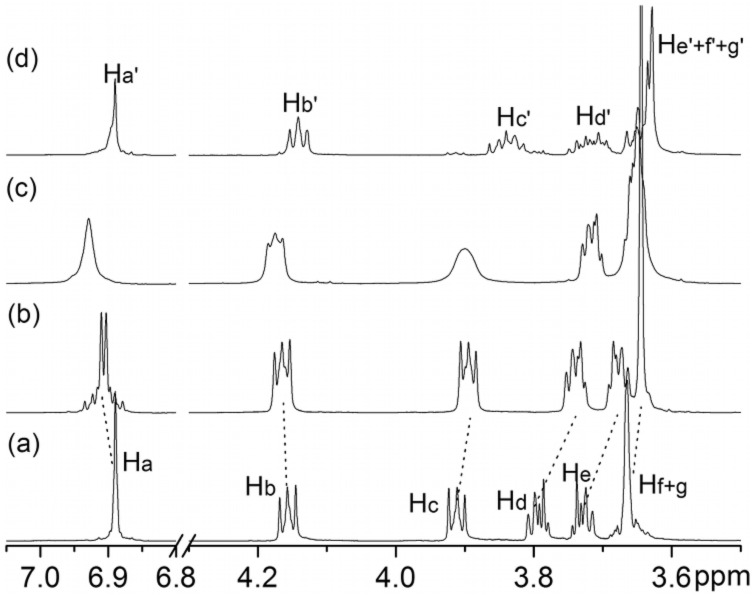
^1^H NMR spectra (500 MHz, 298 K, CDCl_3_, 10.0 mM) of (**a**) **C7**, (**b**) **C7** in the presence of 1 eq. KPF_6_, (**c**) the compound obtained after column chromatography from the KPF_6_-templated reaction, and (**d**) **2**-(*n*).

**Characterization of (C7+KPF_6_) formed in the KPF_6_-templated synthesis of C7.** The ^1^H NMR spectrum (Figure 3c) of the **C7** product obtained from the KPF_6_-templated reaction through extraction with dichloromethane (DCM) from water and column chromatography (eluent gradient: ethylacetate:methanol = 50:1 to 20:1) clearly indicates the formation of a potassium complex which even survived the column chromatography. A comparison with the spectrum of pure **C7** (Figure 3a) and a mixture of pure **C7** and KPF_6_ (Figure 3b) reveals that the product obtained from the column shows similar signal shifts as compared to those of the KPF_6_ complex. This is supported by ESI-MS experiments. In the ESI mass spectrum (Figure S1, Supporting Information) of (**C7**+KPF_6_) sprayed from DCM, three intense peaks at *m/z* 379, 395, and 935 are observed, which can be assigned to [**C7**+Na]^+^, [**C7**+K]^+^ and [**C7**_2_+K+KPF_6_]^+^, respectively. Since no KPF_6_ was added to the solution after column chromatography, the presence of the latter two signals indicated survival of the (**C7**+KPF_6_) complex.

**Figure 4:**
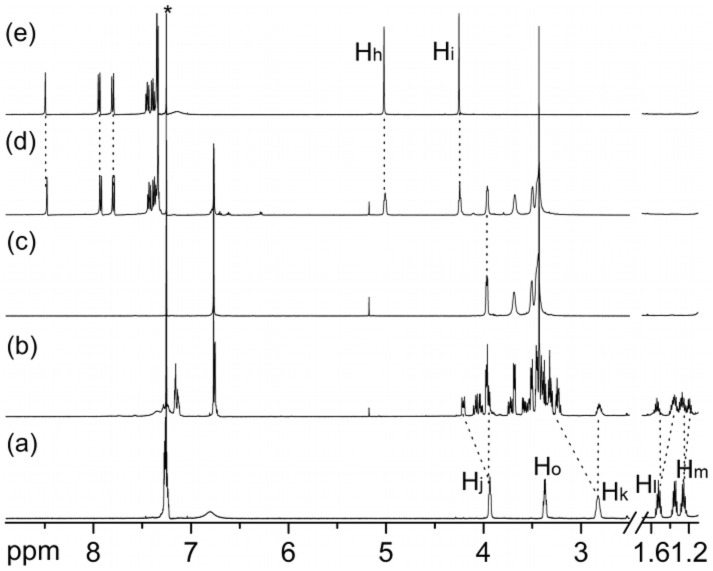
^1^H NMR spectra (500 MHz, 298 K, CDCl_3_:CD_3_CN = 2:1, 10.0 mM) of (**a**) **6**-HoPF_6_, (**b**) mixture of **6**-HoPF_6_ and (**C7**+KPF_6_), (**c**) (**C7**+KPF_6_), (**d**) mixture of **5**-HoPF_6_ and (**C7**+KPF_6_), (**e**) **5**-HoPF_6_. Asterisk = solvent.

Addition of axle **5**-H•PF_6_ to (**C7**+KPF_6_) caused no obvious ^1^H NMR signal changes of one of the building blocks, **5**-H•PF_6_ and (**C7**+KPF_6_) (Figure 4). Axle **5**-H•PF_6_ is consequently not able to replace the potassium ion in (**C7**+KPF_6_) likely because it cannot thread through the cavity.

In marked contrast, the ^1^H NMR spectrum (Figure 4) of a mixture of **6**-H•PF_6_ and (**C7**+KPF_6_) shows a set of new complexation-induced signals, which appear at the same positions as those of independently generated [**6**-H@**C7**]•PF_6_, suggesting that the thinner axle can thread into the crown ether to form the pseudorotaxane even in competition with the potassium ion. This conclusion is further supported by the formation of a white precipitate (KPF_6_) after addition of axle **6**-H•PF_6_ to the (**C7**+KPF_6_) solution in 2:1 CDCl_3_/CD_3_CN. Furthermore, only one intense peak for [**6**-H**@C7**]^+^ is observed in the ESI mass spectrum (Figure S2, Supporting Information). (**C7**+KPF_6_) is sticky solid-like compound rather than oily product [28] as pure **C7** synthesized from **1**. The complex of (**C7**+KPF_6_) could even dissolve in CDCl_3_.

These results demonstrate the difficulties to remove KPF_6_ from **C7** with a standard work-up procedure followed by column chromatography. Considering the good solubility of **C7** in water, more intense washing with water to remove the KPF_6_ salt will likely reduce the yield.

Quite interestingly, the use of KBF_4_ as the template during the synthesis of **C7** from catechol and **3** results in a much more easily achievable separation of uncomplexed **C7**. We speculate that the lower solubility of this salt in organic solvent helps to separate the crown ether from the salt during the extraction.

**The effect of substituents on binding affinity and binding mode.** The binding of axles **6**-H•PF_6_ and **7**-H•PF_6_ to **C7** is a slow process on the NMR time scale. Consequently, the corresponding binding constants of [**6**-H@**C7**]•PF_6_, [**6**-H@**4**]•PF_6_, [**7**-H@**C7**]•PF_6_, and [**7**-H@**4**]•PF_6_ in 2:1 CDCl_3_/CD_3_CN solution (Figures S3–S10, Supporting Information) can easily be determined from the total host concentration and the relative integration of the separate signals for free and complexed hosts [31]. They are 17090 (±500) M^−1^, 8000 (±270) M^−1^, 5640 (±190) M^−1^, and 3050 (±60) M^−1^, respectively. The lower binding ability of **4** relative to **C7** is certainly due to the electron-withdrawing aldehyde group which decreases the electron-donating and hydrogen-bond accepting ability of the oxygen atoms on the catechol [32]. Consequently, electron-withdrawing substitution on **C7** should be avoided when aiming at strong binding between the two building blocks.

Literature reports that a change of guest from secondary dibenzylammonium hexafluorophosphate (360 M^−1^, 1.0 mM, in acetone-*d*_6_) [31] to the anthracenyl methyl-substituted analogue **5**-H•PF_6_ (496 M^−1^, 1.0 mM, in acetone-*d*_6_) [26] increases the binding affinity with DB24C8, which is mainly attributed to stronger π-π stacking interactions with the larger anthracene π-system in **5**-H•PF_6_.

**Figure 5:**
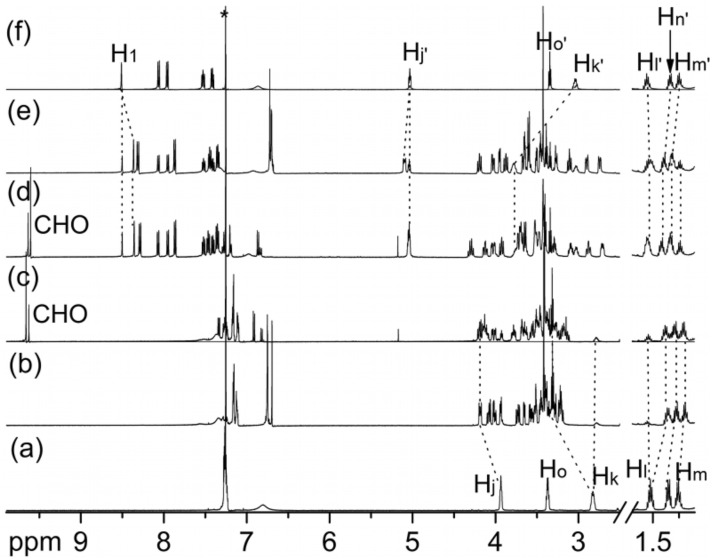
^1^H NMR spectra (500 MHz, 298 K, CDCl_3_:CD_3_CN = 2:1, 10.0 mM) of (**a**) **6**-H•PF_6_, equimolar mixtures of (**b**) **6**-H•PF_6_ and **C7**, (**c**) **6**-H•PF_6_ and **4**, (**d**) **7**-H•PF_6_ and **4**, and (**e**) **7**-H•PF_6_ and **C7**, and (**f**) **7**-H•PF_6_, Asterisk = solvent residue.

Analogously, stronger binding of **C7** would be expected with **7**-H•PF_6_ as compared to **6**-H•PF_6_. Surprisingly, the binding affinities of **C7** or **4** toward anthracenyl methyl-substituted **7**-H•PF_6_ turn out to be lower than to benzyl-substituted **6**-H•PF_6_. There are two reasons for this remarkable difference between **C7** and the larger analogue dibenzo-24-crown-8. (i) According to related crystal structures [23–25], no π-π stacking interactions operate between hosts **C7** or **4** and guests **6**-H•PF_6_ or **7**-H•PF_6_. (ii) Even more important, however, are the polarized methylene groups next to the ammonium center. These groups form C-H•••O hydrogen bonds [33] with the crown ether as indicated by the quite substantial complexation-induced downfield shifts (0.25 and 0.55 ppm, respectively, observed for H_j_ and H_k_ of [**6**-H@**C7**]•PF_6_ and [**6**-H@**4**]•PF_6_ (Figure 5b,Figure 5c) relative to free **6**-H•PF_6_. In contrast, H_j′_ on **7**-H•PF_6_ is observed to shift downfield by only 0.05 ppm after complexation with **C7** and undergoes hardly any shift when the axle is complexed to **4**, while H_k′_ experiences a 0.76 ppm upfield shift for complexing with both hosts (Figure 5d,Figure 5e). These facts suggests that H_j’_ of **7**-H•PF_6_ may be only loosely involved in the C-H•••O hydrogen-bonding with **C7** or **4** due to the increased steric demand of the anthracenyl methyl group. Consequently, the symmetry and the cavity size of dibenzo-24-crown-8 are suitable to adopt to the requirements of the anthracenyl methyl group and the binding energy increases, when phenyl is replaced by anthracenyl. Instead, the cavity of **C7** is smaller and likely unable to adjust itself to the anthracenyl methyl-substituted axle. Some of the C-H•••O hydrogen bonds which can form with **6**-H•PF_6_ do not form with **7**-H•PF_6_ and thus weaken the complexes of the latter axle.

## Conclusion

In summary, two procedures have been explored for the synthesis of **C7**. The one with catechol and hexa(ethylene glycol) ditosylate as starting materials and KBF_4_ as template turned out to be a quite efficient synthetic pathway allowing easy introduction of a variety of substituents by choosing the appropriate catechol building block. In addition, two guests **5**-H•PF_6_ and **6**-H•PF_6_ are found to be very useful for the characterization of **C7** and its homologues on the basis of the fact that **C7** could not pass over phenyl group. Modifications of **C7** and secondary dialkylammonium guests significantly alter the binding ability. Replacing a benzyl stopper on the axle by an anthracenyl methyl group even changes the binding mode: Formation of C-H•••O hydrogen bonds is hampered for the methylene group between the anthracene and the ammonium. Compared to DB24C8, the complexing property of **C7** is more susceptible to modification probably because the smaller macrocycle is more or less rigidified after complexation with secondary dialkylammonium, thus weakening its adjustability. This has to be taken into account if one desires to construct more complex interlocked assemblies by using **C7** and secondary dialkylammonium ions as building blocks in the future.

## Experimental

**General Methods.** All reagents were commercially available unless explicitly stated and used without further purification. 1,2-Bis{2-[2-(2-hydroxyethoxy)ethoxy]ethoxy}benzene [34], **5**-H•PF_6_ [35] and **6**-H•PF_6_ [23] were synthesized according to literature procedures. Solvents were either employed as purchased or dried prior to use by usual laboratory methods. Thin-layer chromatography (TLC) was performed on aluminum sheets coated with silica gel 60/F_254_ (Merck KGaA). The plates were inspected by UV light, and if required, developed in I_2_ vapor. Column chromatography was performed on silica gel 60 (Merck 40–60 nm, 230–400 mesh). ^1^H and ^13^C NMR spectra were recorded on Bruker ECX 400 MHz and Jeol Eclipse 500 MHz. All chemical shifts are reported in ppm with residual solvents as the internal standards, and the coupling constants (*J*) are in Hertz. The following abbreviations were used for signal multiplicities: s, singlet; d, doublet; t triplet; m, multiplet. Electrospray-ionization time-of-flight high-resolution mass spectrometry (ESI-TOF-HRMS) experiments were conducted on an Agilent 6210 ESI-TOF, Agilent Technologies and a Varian/IonSpec QFT-7 FTICR (Fourier-transform ion-cyclotron-resonance) mass spectrometer equipped with a superconducting 7 Tesla magnet and a micromass Z-spray Electrospray-ionization (ESI) ion source utilizing a stainless steel capillary with a 0.75 mm inner diameter.

**2-{2-[2-(2-{2-[2-(2-Hydroxyethoxy)ethoxy]ethoxy}phenoxy)ethoxy]ethoxy}ethyl-4-methylbenzene-sulfonate (1):** To a mixture of 1,2-Bis{2-[2-(2-hydroxyethoxy)ethoxy]ethoxy}benzene (5.15 g, 13.8 mmol) in THF (60 mL) and sodium hydroxide (2.2 g, 55 mmol) in H_2_O (60 mL) in an ice bath was added dropwise tosyl chloride (3.2 g, 16.8 mmol) in THF (150 mL) for 2 h. The mixture was continued to stir overnight in ice bath, THF was evaporated under reduced pressure. The residue was suspended in H_2_O (50 mL), extracted with CH_2_Cl_2_ (100 mL × 3) and then dried over anhydrous Na_2_SO_4_. After the solvent was removed in vacuo, the crude product was subjected to column chromatography (silica gel, eluent: ethyl acetate: hexane = 2:1) to afford a pale-yellow oil **1** (3.0 g, 41%). ^1^H NMR (400 MHz, CDCl_3_, 298 K): δ (ppm) = 2.42 (s, 3H), 3.58–3.62 (m, 4H), 3.64–3.75 (m, 10H), 3.80–3.88 (m, 4H), 4.12–4.17 (m, 6H), 6.89–6.91 (m, 4H), 7.31 (d, *J* = 8.0 Hz, 2H), 7.78 (d, *J* = 8.4 Hz, 2H); ^13^C NMR (100 MHz, CDCl_3_, 298 K): δ (ppm) = 21.7, 61.9, 68.8, 68.91, 68.94, 69.4, 69.89, 69.90, 70.5, 70.8, 70.9, 71.0, 72.6, 115.0, 121.8, 128.0, 129.9, 133.0, 144.9, 149.0; ESI-TOF-HRMS: *m/z* calcd for [M+Na]^+^ (100%): 551.1921, found: 551.1926; *m/z* calcd for [M+K]^+^ (20%): 567.1661, found: 567.1664.

**Benzo-21-crown-7 (C7) and its homologues (2-(*n*)):** The mixture of **1** (2.37 g, 4.5 mmol) and NaH (0.60 g, 25.0 mmol) in anhydrous THF (50 mL) was refluxed for 3 d. After cooling down to room temperature, water (100 mL) was added to quench the superfluous NaH. THF was removed under reduced pressure, and the residue was extract by CH_2_Cl_2_ (100 mL × 3). The organic phase was collected, dried over anhydrous Na_2_SO_4_, and concentrated in vacuo to give the crude product, which was isolated by column chromatography (silica gel, eluent: ethyl acetate/MeOH, 100:1 to 20:1) to afford **C7** [23,28] (380 mg, 24%) and **2**-(*n*) (490 mg, 31%) as yellow oil. For **C7**, ^1^H NMR (400 MHz, CDCl_3_, 298 K): δ (ppm) = 3.64–3.69 (m, 8H), 3.71–3.75 (m, 4H), 3.77–3.81 (m, 4H), 3.92 (t, *J* = 4.6 Hz, 4H), 4.16 (t, *J* = 4.6 Hz, 4H), 6.87–6.91 (m, 4H); ^13^C NMR (100 MHz, CDCl_3_, 298 K): δ (ppm) = 69.3, 69.9, 70.6, 71.07, 71.13, 71.16, 114.5, 121.6, 149.0; For **2**-(*n*), ^1^H NMR (400 MHz, CDCl_3_, 298 K): δ (ppm) = 3.57–3.68 (m, 12(*n*+1)H), 3.68–3.76 (m, 4(*n*+1)H), 3.79–3.87 (m, 4(*n*+1)H), 4.12–4.18 (m, 4(*n*+1)H), 6.86–6.94 (m, 4(*n*+1)H); ^13^C NMR (100 MHz, CDCl_3_, 298 K): δ (ppm) = 68.9, 69.0, 69.1, 69.8, 69.9, 70.6, 70.66, 70.71, 70.75, 70.87, 70.89, 70.93, 71.08, 71.14, 71.17, 114.8, 115.0, 121.6, 121.7, 149.1.

**Hexa(ethylene glycol) ditosylate (3):** Hexa(ethylene glycol) (5.0 g, 17.7 mol) in THF (50 mL) and sodium hydroxide (4.8 g, 120 mmol) in H_2_O (50 mL) was mixed in 500 mL flask. To the mixture in an ice bath was added dropwise tosyl chloride (12 g, 63 mmol) in THF (100 mL) for 2 h. The reaction mixture was stirred for another 5 h in ice bath, and THF was then concentrated under reduced pressure. The residue was suspended in H2O (150 ml) and extracted with dichloromethane (100 mL × 3) and then dried over anhydrous Na_2_SO_4_. The solvent was removed in vacuo to give **3** [23] as a pale-yellow oil (10 g, 96%) which is pure enough for next step. ^1^H NMR (400 MHz, CDCl_3_, 298 K): δ (ppm) = 2.44 (s, 6H), 3.55–3.64 (m, 16H), 3.67 (t, *J* = 4.8 Hz, 4H), 4.14 (t, *J* = 4.8 Hz, 4H), 7.33 (d, *J* = 8.0 Hz, 2H), 7.79 (d, *J* = 8.0 Hz, 2H).

**General procedure for synthesis of C7 KPF_6_ or KBF_4_ as template and 4 with KBF_4_ as template:** While stirring vigorously under argon atmosphere, a suspension of K_2_CO_3_ (2.07 g, 15 mmol) and KPF_6_ or KBF_4_ (7.5 mmol) in anhydrous CH_3_CN (100 mL) was heated to reflux. To the suspension was added dropwise a solution of **3** (2.95 g, 5.0 mmol) and catechol or 3,4-dihydroxybenzaldehyde (5.0 mmol) in CH_3_CN (100 mL) during 12 h. The resulting reaction mixture was stirred under reflux for another 3 d. Upon cooling down to ambient temperature, the suspension was filtered and washed with CH_2_Cl_2_ (100 mL). The filtrate was concentrated under vacuum. The residue was partitioned between CH_2_Cl_2_ (100 mL) and water (100 mL), and the aqueous phase was extracted twice by CH_2_Cl_2_ (50 mL). The combined organic phase was dried over anhydrous Na_2_SO_4_, and concentrated under reduced pressure to give the crude product, which was purified by column chromatography over silica gel (eluent: ethyl acetate/MeOH, from 50:1 to 20:1). For **C7** (with KBF_4_ as template) (1.25 g, 70%), yellow oil, the ^1^H NMR spectrum is in line with the literature [23,28] and the one synthesized from compound **1**; For **4** (1.20 g, 62%), yellow oil; ^1^H NMR (400 MHz, CDCl_3_, 298 K): δ (ppm) = 3.63–3.69 (m, 8H), 3.70–3.75 (m, 4H), 3.77–3.82 (m, 4H), 3.91–3.97 (m, 4H), 4.18–4.24 (m, 4H), 6.95 (d, *J* = 8.4 Hz, 1H), 7.38 (d, *J* = 1.6 Hz, 1H), 7.43 (dd, *J_1_* = 8.4 Hz, *J_2_* = 1.6 Hz, 1H), 9.82 (s, 1H); ^13^C NMR (100 MHz, CDCl_3_, 298 K): δ (ppm) = 69.2, 69.3, 69.5, 69.6, 70.6, 71.0, 71.05, 71.1, 71.2, 71.3, 71.4, 111.4, 112.3, 126.9, 130.3, 149.2, 154.4, 190.9; ESI-TOF-HRMS: *m/z* calcd for [M+K]^+^ (100%): 423.1416, found: 423.1434.

**5-[(Anthracen-10-yl)methylamino]pentan-1-ol (7):** 9-Anthracenecarboxaldehyde (1.00 g, 4.9 mmol) and 5-aminopentan-1-ol (0.71 mL, 6.5 mmol) were refluxed for 24 h in a mixture of 90 ml of absolute ethanol and 60 ml of CHCl_3_. After cooling down to room temperature, NaBH_4_ (1.86 g, 49 mmol) was added and the resulting solution stirred at room temperature for another 24 h. The solvent was removed under vacuum. The resulting residue was treated with water and the compound was repeatedly extracted with CH_2_Cl_2_ (three times 50 ml). The organic phase was dried over anhydrous Na_2_SO_4_, and the solvent was evaporated to give the crude product, which was subjected to column chromatography over silica gel (eluent, CH_2_Cl_2_:MeOH, 100:1 to 20:1) to afford **7** [36] (1.00 g, 70%) as a yellow solid. ^1^H NMR (400 MHz, CDCl_3_, 298 K): δ (ppm) = 1.37–1.46 (m, 2H), 1.51–1.65 (m, 4H), 2.87 (t, *J* = 7.0 Hz, 2H), 3.59 (t, *J* = 6.4 Hz, 2H), 4.73 (s, 2H), 7.43–7.48 (m, 2H), 7.51–7.56 (m, 2H), 7.98–8.02 (m, 2H), 8.30–8.35 (m, 2H), 8.40 (s, 1H); ^13^C NMR (100 MHz, CDCl_3_, 298 K): δ (ppm) = 23.5, 29.7, 32.6, 45.8, 50.4, 62.8, 124.2, 125.0, 126.2, 129.3, 130.4, 131.6; ESI-TOF-HRMS: *m/z* calcd for [M+H]^+^ (100%): 294.1852, found: 294.1858.

**7-H**•**PF_6_:** To compound **7** (1.00 g, 3.41 mmol) dissolved in MeOH (30 mL) was added conc. HCl to adjust pH < 2, and the solvent was then evaporated off under reduced pressure. The residue was suspended in acetone (30 mL). Saturated aqueous NH_4_PF_6_ solution was added until the suspension became clear. The solvent was removed in vacuo, and water (100 mL) was added to the residue. The resulting mixture was stirred at ambient temperature overnight. The mixture was then filtered, washed with copious amounts of H_2_O, and dried to give **7**-H•PF_6_ as a yellow solid (1.39 g, 92%). ^1^H NMR (400 MHz, CD_3_CN, 298 K): δ (ppm) = 1.36–1.44 (m, 2H), 1.46–1.54 (m, 2H), 1.69–1.78 (m, 2H), 3.25–3.34 (m, 2H), 3.48 (t, *J* = 6.2 Hz, 2H), 5.23 (t, *J* = 6.2 Hz, 2H), 7.58–7.64 (m, 2H), 7.70–7.76 (m, 2H), 8.14–8.19 (m, 2H), 8.30–8.34 (m, 2H), 8.74 (s, 1H); ^13^C NMR (100 MHz, CD_3_CN, 298 K): δ (ppm) = 23.4, 26.2, 32.3, 44.9, 49.9, 62.0, 122.0, 124.2, 126.6, 128.6, 130.4, 131.8, 132.3; ESI-TOF-HRMS: *m/z* calcd for [M-PF_6_]^+^ (100%): 294.1852, found: 294.1852.

## Supporting Information

File 1 NMR and MS spectra of the corresponding complexes.

## References

[1] Sauvage J-P, Dietrich-Buchecker C O (1999). Molecular Catenanes, Rotaxanes and Knots.

[2] Stoddart J F, Colquhoun H M (2008). Tetrahedron.

[3] Stoddart J F (2009). Chem Soc Rev.

[4] Stoddart J F (2009). Chem Soc Rev.

[5] Balzani V, Credi A, Raymo F M, Stoddart J F (2000). Angew Chem.

[6] Stoddart J F (2001). Molecular Machines Special Issue In. Acc Chem Res.

[7] Feringa B L (2001). Molecular Switches.

[8] Balzani V, Venturi M, Credi A (2003). Molecular Devices and Machines – A Journey into the Nano World.

[9] Kay E R, Leigh D A, Zerbetto F (2007). Angew Chem.

[10] Schill G, Zollenkopf H (1969). Justus Liebigs Ann Chem.

[11] Hiratani K, Suga J, Nagawa Y, Houjou H, Tokuhisa H, Numata M, Watanabe K (2002). Tetrahedron Lett.

[12] Hiratani K, Albrecht M (2008). Chem Soc Rev.

[13] Schalley C A, Vögtle F, Dötz K-H (2004). Templates in Chemistry I. Top Curr Chem.

[14] Schalley C A, Vögtle F, Dötz K-H (2005). Templates in Chemistry II. Top Curr Chem.

[15] Broekmann P, Dötz K-H, Schalley C A (2009). Templates in Chemistry III. Top Curr Chem.

[16] Schalley C A, Illigen J, Ariga K, Nalwa H S (2009). Templated Synthesis of Interlocked Molecules. Bottom-up Nanofabrication: Supramolecules, Self-Assemblies, and Organized Films.

[17] Kolchinski A G, Busch D H, Alcock N W (1995). J Chem Soc, Chem Commun.

[18] Kolchinski A G, Alcock N W, Roesner R A, Busch D H (1998). Chem Commun.

[19] Gibson H W, Yamaguchi N, Hamilton L, Jones J W (2002). J Am Chem Soc.

[20] Huang F-H, Jones J W, Gibson H W (2007). J Org Chem.

[21] Wu J, Leung K C-F, Stoddart J F (2007). Proc Natl Acad Sci U S A.

[22] Wu J, Leung K C-F, Benítez D, Han J-Y, Cantrill S J, Fang L, Stoddart J F (2008). Angew Chem.

[23] Zhang C-J, Li S-J, Zhang J-Q, Zhu K-L, Li N, Huang F-H (2007). Org Lett.

[24] Zhang C-J, Zhu K-L, Li S-J, Zhang J-Q, Wang F, Liu M, Li N, Huang F-H (2008). Tetrahedron Lett.

[25] Hsu C-C, Chen N-C, Lai C-C, Liu Y-H, Peng S-M, Chiu S-H (2008). Angew Chem.

[26] Jiang W, Winkler H D F, Schalley C A (2008). J Am Chem Soc.

[27] Jiang W, Schalley C A (2009). Proc Natl Acad Sci U S A.

[28] Bogaschenko T, Basok S, Kulygina C, Lyapunov A, Lukyanenko N (2002). Synthesis.

[29] Ostrowicki A, Koepp E, Vögtle F (1992). The “cesium effect”: Syntheses of medio- and macrocyclic compounds. Top. Curr. Chem.

[30] Leize E, Jaffrezic A, Van Dorsselaer A (1996). J Mass Spectrom.

[31] Ashton P R, Chrystal E J T, Glink P, Menzer S, Schiavo C, Spencer N, Stoddart J F, Tasker P A, White A J P, Williams D J (1996). Chem–Eur J.

[32] Liu Y, Li C-J, Zhang H-Y, Wang L-H, Li X-Y (2007). Eur J Org Chem.

[33] Ashton P R, Campbell P J, Chrystal E J T, Glink P, Menzer S, Philp D, Spencer N, Stoddart J F, Tasker P A, Williams D J (1995). Angew Chem.

[34] Jiang W, Han M, Zhang H-Y, Zhang Z-J, Liu Y (2009). Chem–Eur J.

[35] Ashton P R, Ballardini R, Balzani V, Gómez-López M, Lawrence S E, Martínez-Díaz M V, Montalti M, Piersanti A, Prodi L, Stoddart J F (1997). J Am Chem Soc.

[36] Clifford T, Abushamleh A, Busch D H (2002). Proc Natl Acad Sci U S A.

