# Supplementary material for: Templated versus non-templated synthesis of benzo-21-crown-7 and the influence of substituents on its complexing properties
Source: Beilstein J Org Chem. 2010 Feb 11;6:14. doi: 10.3762/bjoc.6.14 (PMC2869786; doi:10.3762/bjoc.6.14)
Supplement: File 1 — NMR and MS spectra of the corresponding complexes. [file Beilstein_J_Org_Chem-06-14-s001.pdf]

# S1: NMR and MS spectra of the corresponding complexes

## Supporting Information

**for**

## Templated versus non-templated synthesis of benzo-21-crown-7 and the influence of substituents on its complexing properties

Wei Jiang and Christoph A. Schalley*

Address: Institut für Chemie und Biochemie, Freie Universität Berlin, Takustraße 3, 14195 Berlin, Germany

E-mail: Christoph A. Schalley - christoph@schalley-lab.de

* Corresponding author

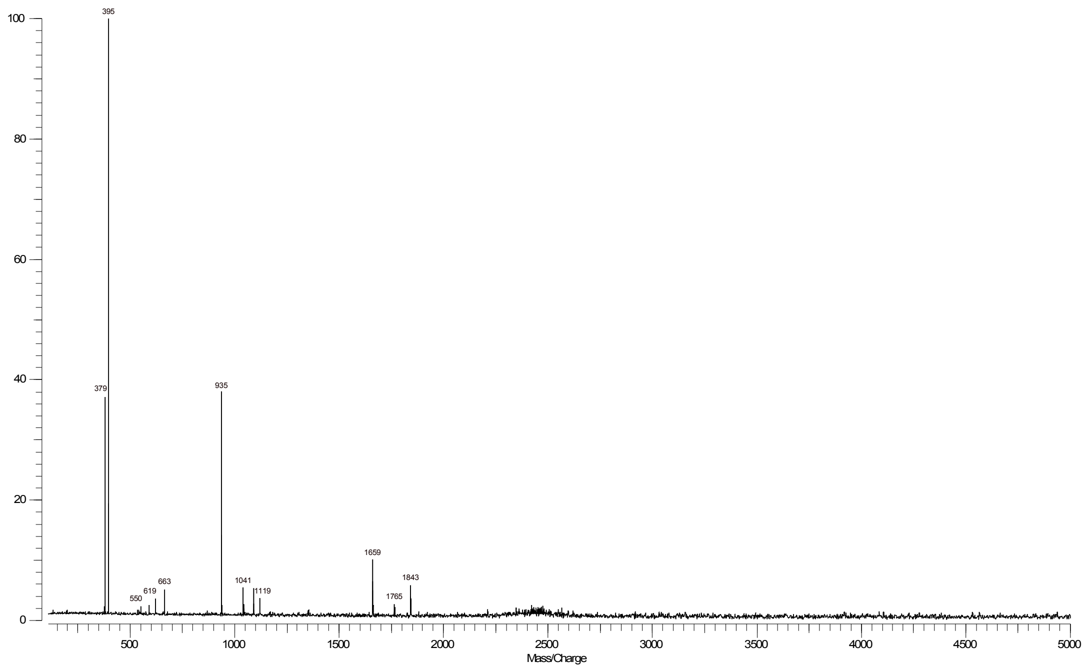


**Figure S1:** ESI-FTICR Mass spectrum of (**C7**+$KPF_6$) sprayed from DCM. The peaks at *m/z* 379, 395, and 935 are assigned to [**C7**+Na]$^+$, [**C7**+K]$^+$, and [2**C7**+K+$KPF_6$]$^+$, respectively. Since we didn't deliberately add $KPF_6$ into this solution, $KPF_6$ should be from the template used in the reaction which could not be removed after extraction and column chromatography. This result is in agreement with NMR results.

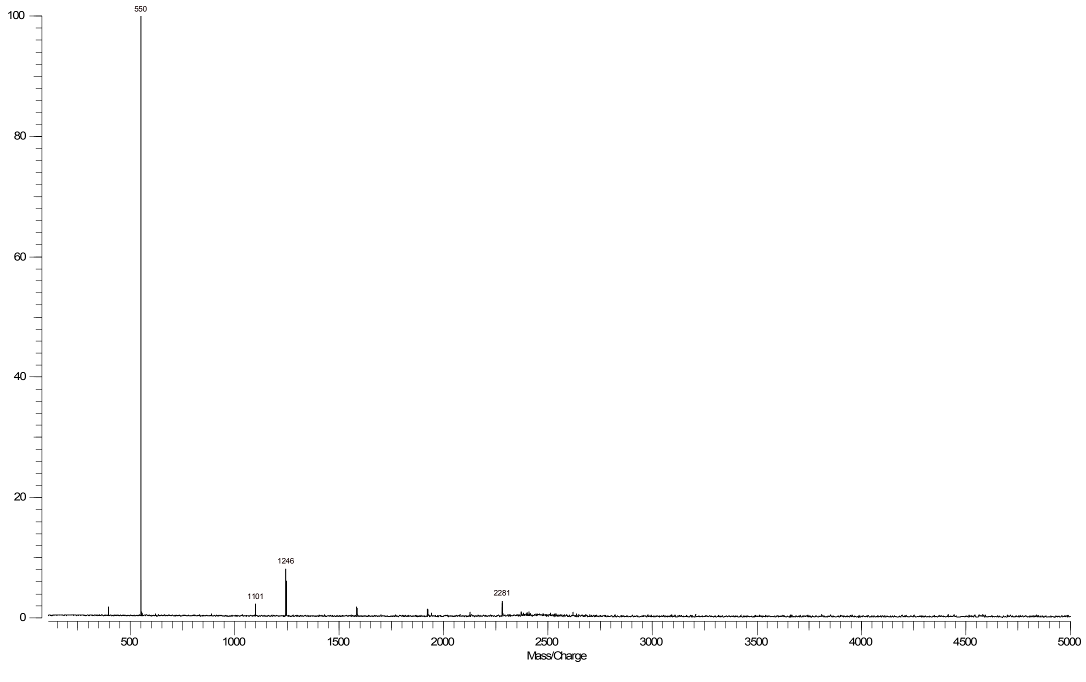


**Figure S2:** ESI-FTICR Mass spectrum of (**C7**+$KPF_6$) in the presence of **6**-H·$PF_6$. The peak at *m/z* 550 is assigned to [**6**-H@**C7**]$^+$. The only intense peak suggests that **C7** is the dominant organic compound in (**C7**+$KPF_6$) and **6**-H·$PF_6$ fits better to **C7** than $KPF_6$.

**The association constants which were calculated by single-point method:**

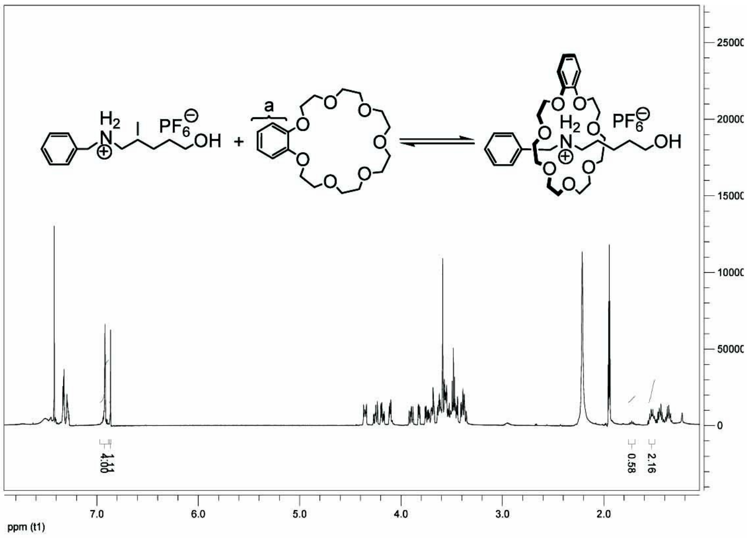


**Figure S3:** $^{1}H$ NMR spectrum (500 MHz, 298 K, $CDCl_3$:$CD_3CN$ = 2:1, 1.0 mM) of the equimolar mixture of **6**-H·$PF_6$ and **C7**. From complexed and uncomplexed $H_a$ of **C7**, $K_a(H_a)$ = [(4.00/5.11) × 1.0 × $10^{-3}$] / [(1.11/5.11) × 1.0 × $10^{-3}$]$^2$ $M^{-1}$ = 16590 $M^{-1}$; From complexed and uncomplexed $H_l$ of **6**-H·$PF_6$, $K_a(H_l)$ = [(2.16/2.74) × 1.0 × $10^{-3}$] / [(0.58/2.74) × 1.0 × $10^{-3}$]$^2$ $M^{-1}$ = 17590 $M^{-1}$. Finally, $K_a$ = (16590+17590)/2 = 17090 (±500) $M^{-1}$.

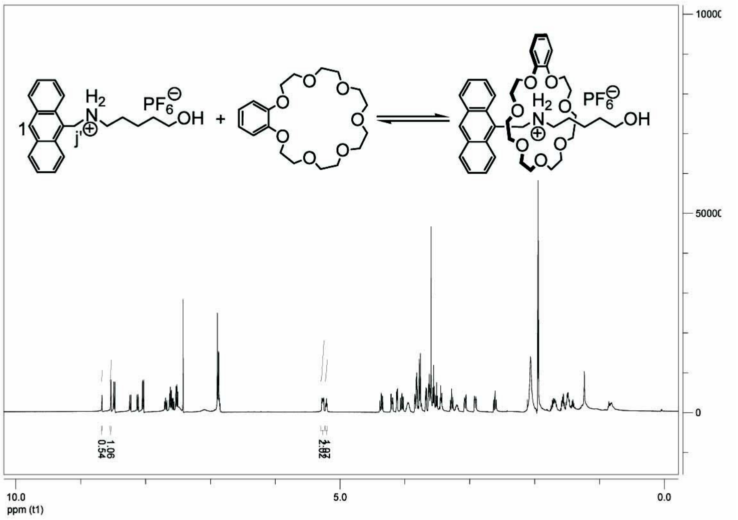


**Figure S4:** $^{1}H$ NMR spectrum (500 MHz, 298 K, $CDCl_3$:$CD_3CN$ = 2:1, 1.0 mM) of the equimolar mixture of **7**-H·$PF_6$ and **C7**. From complexed and uncomplexed $H_1$ of **7**-H·$PF_6$, $K_a(H_1)$ = [(1.06/1.60) × 1.0 × $10^{-3}$] / [(0.54/1.60) × 1.0 × $10^{-3}$]$^2$ $M^{-1}$ = 5820 $M^{-1}$; From complexed and uncomplexed $H_{j'}$ of **7**-H·$PF_6$, $K_a(H_{j'})$ = [(2.02/3.09) × 1.0 × $10^{-3}$] / [(1.07/3.09) × 1.0 × $10^{-3}$]$^2$ $M^{-1}$ = 5450 $M^{-1}$. Finally, $K_a$ = (5820+5450)/2 = 5640 (±190) $M^{-1}$.

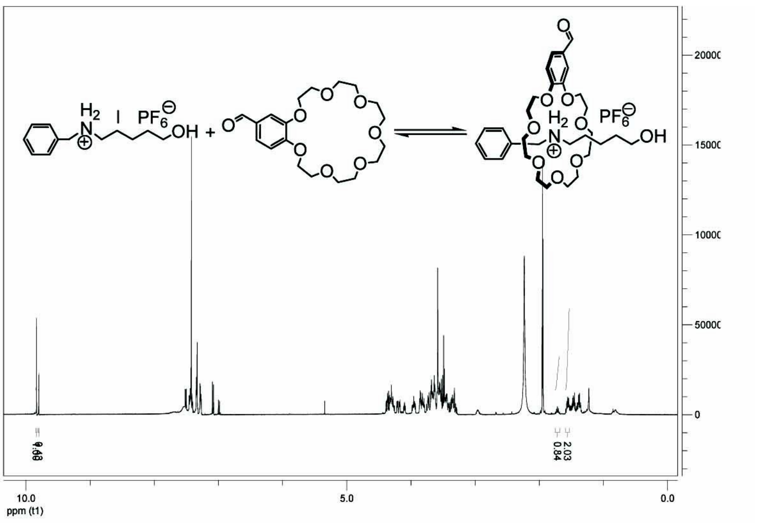


**Figure S5:** $^1$H NMR spectrum (500 MHz, 298 K, $CDCl_3$:$CD_3CN$ = 2:1, 1.0 mM) of the equimolar mixture of **6**-H·$PF_6$ and **4**. From complexed and uncomplexed CHO of **4**, $K_a$(CHO) = $[(1.00/1.43) \times 1.0 \times 10^{-3}] / [(0.43/1.43) \times 1.0 \times 10^{-3}]^2$ $M^{-1}$ = 7730 $M^{-1}$; From complexed and uncomplexed $H_l$ of **6**-H·$PF_6$, $K_a(H_l) = [(2.03/2.87) \times 1.0 \times 10^{-3}] / [(0.84/2.87) \times 1.0 \times 10^{-3}]^2$ $M^{-1}$ = 8260 $M^{-1}$. Finally, $K_a$ = (7730+8260)/2 = 8000 (±270) $M^{-1}$.

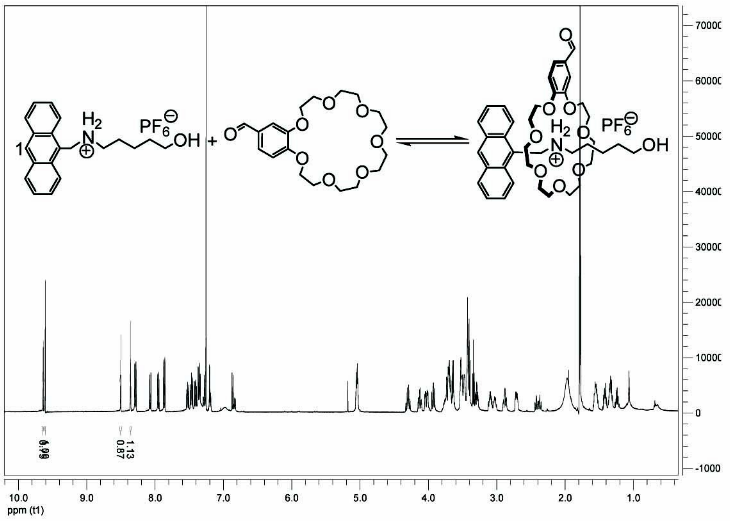


**Figure S6:** $^1$H NMR spectrum (500 MHz, 298 K, $CDCl_3$:$CD_3CN$ = 2:1, 1.0 mM) of the equimolar mixture of **7**-H·$PF_6$ and **4**. From complexed and uncomplexed CHO of **4**, $K_a$(CHO) = $[(1.00/1.75) \times 1.0 \times 10^{-3}] / [(0.75/1.75) \times 1.0 \times 10^{-3}]^2$ $M^{-1}$ = 3110 $M^{-1}$; From complexed and uncomplexed $H_1$ of **7**-H·$PF_6$, $K_a(H_1) = [(1.13/2.00) \times 1.0 \times 10^{-3}] / [(0.87/2.00) \times 1.0 \times 10^{-3}]^2$ $M^{-1}$ = 2990 $M^{-1}$. Finally, $K_a$ = (3110+2990)/2 = 3050 (±60) $M^{-1}$.

**$^1$H-$^1$H COSY Spectra:**

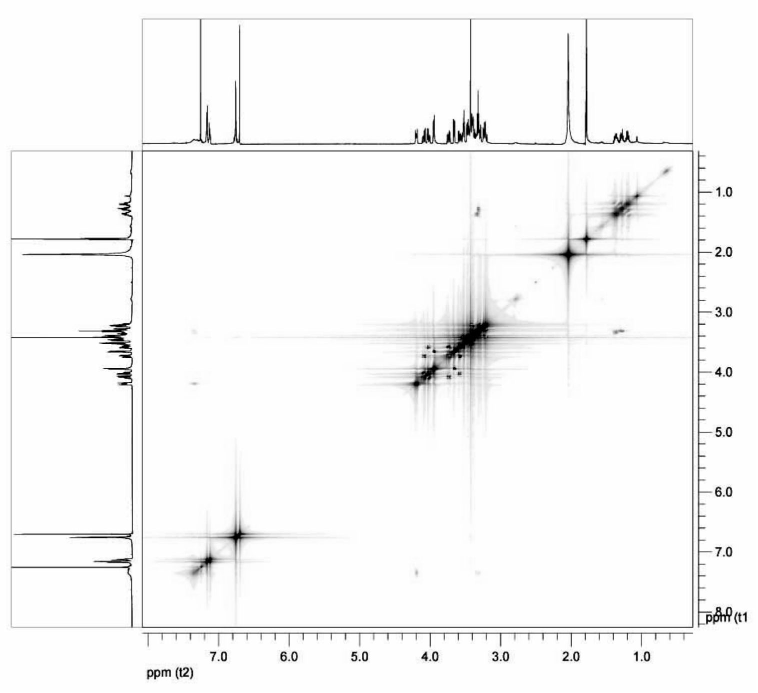


**Figure S7:** $^1$H-$^1$H COSY spectrum (500 MHz, 298 K, $CDCl_3$:$CD_3CN$ = 2:1, 10.0 mM) of the equimolar mixture of **6**-H·$PF_6$ and **C7**. The COSY spectrum supports the assignments of the peaks in Figure 5b.

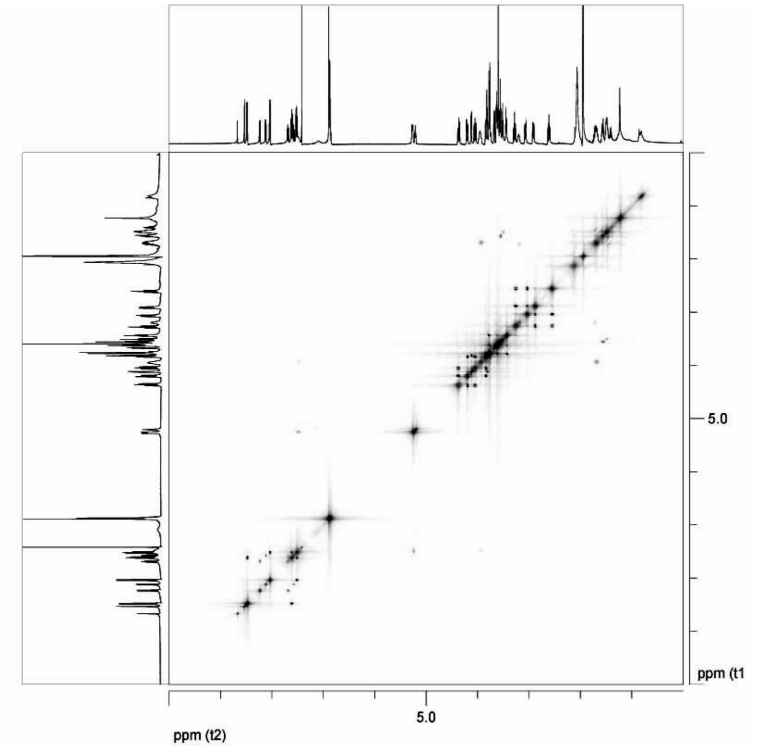


**Figure S8:** $^1$H-$^1$H COSY spectrum (500 MHz, 298 K, $CDCl_3$:$CD_3CN$ = 2:1, 10.0 mM) of the equimolar mixture of **7**-H·$PF_6$ and **C7**. The COSY spectrum supports the assignments of the peaks in Figure 5e.

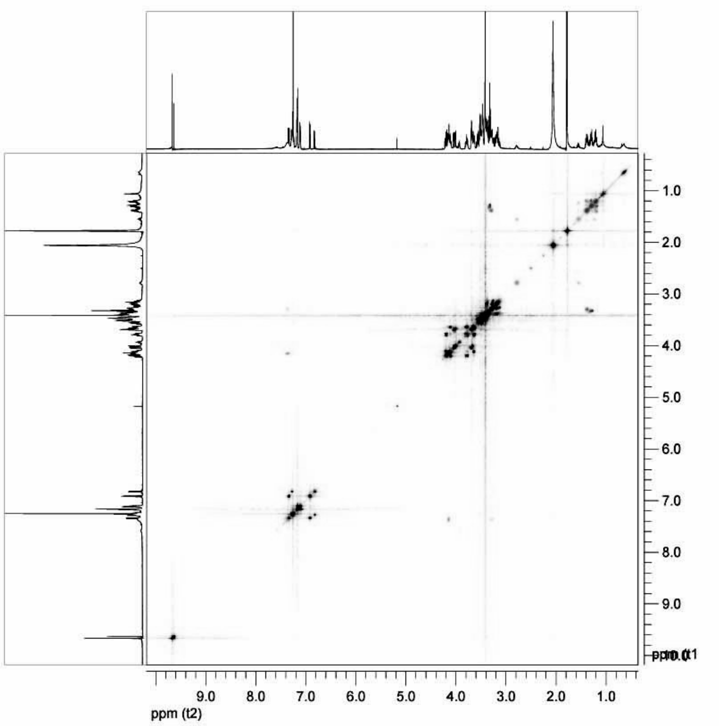


**Figure S9:** $^{1}H$-$^{1}H$ COSY spectrum (500 MHz, 298 K, $CDCl_3$:$CD_3CN$ = 2:1, 10.0 mM) of the equimolar mixture of **6**-H·$PF_6$ and **4**. The COSY spectrum supports the assignments of the peaks in Figure 5c.

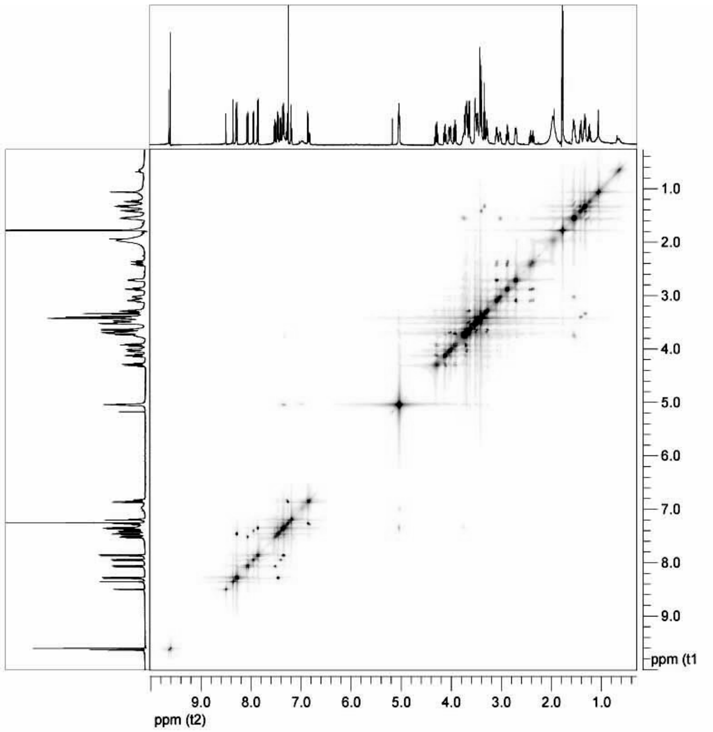


**Figure S10:** $^{1}H$-$^{1}H$ COSY spectrum (500 MHz, 298 K, $CDCl_3$:$CD_3CN$ = 2:1, 10.0 mM) of the equimolar mixture of **7**-H·$PF_6$ and **4**. The COSY spectrum supports the assignments of the peaks in Figure 5d.

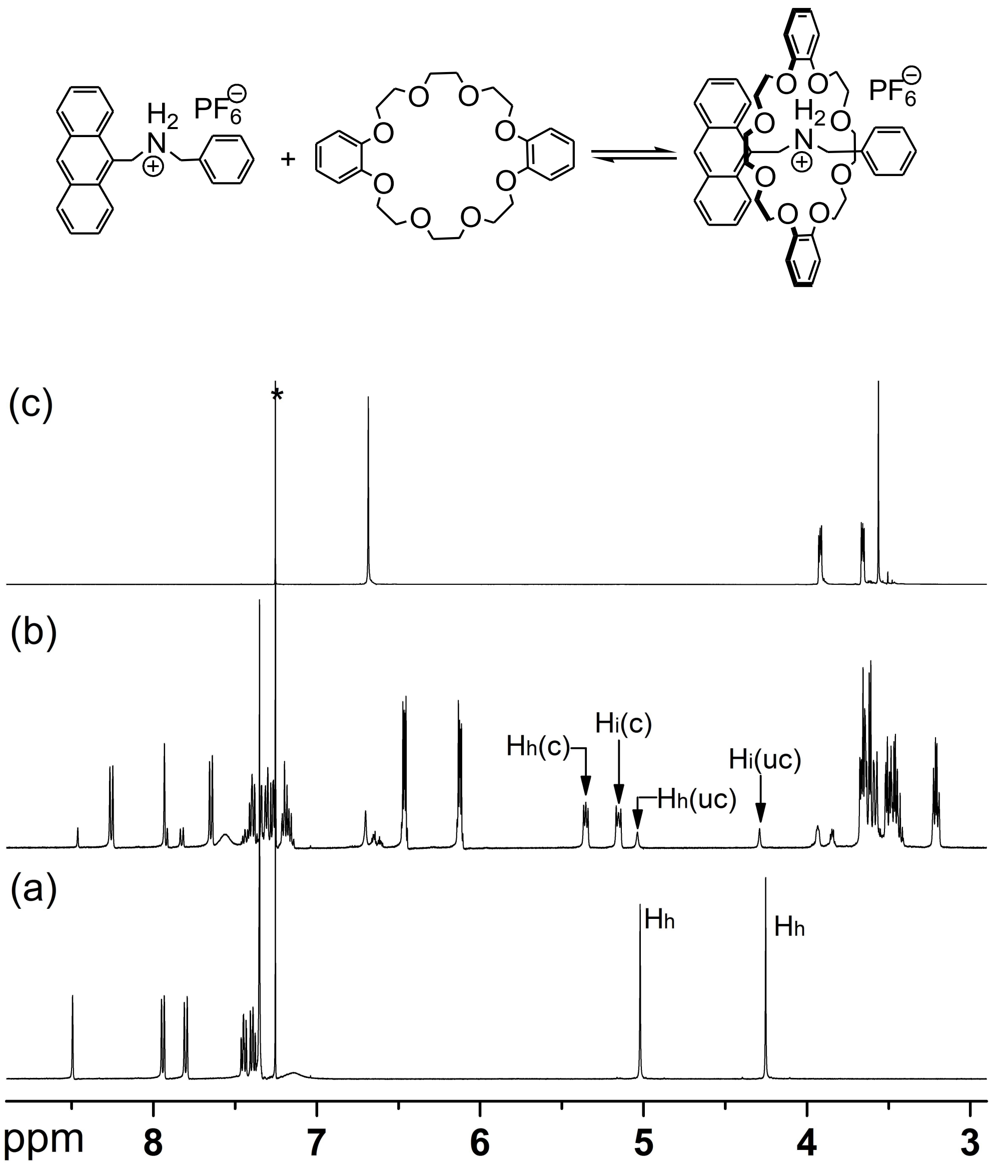


**Figure S11:** Top: complexation of **5**-H·$PF_6$ and DB24C8; Bottom: $^1H$ NMR spectra (500 MHz, 298 K, $CDCl_3$:$CD_3CN$ = 2:1, 10.0 mM) of (a) **5**-H·$PF_6$, (c) DB24C8, and (b) the equimolar mixture of **5**-H·$PF_6$ and DB24C8. Asterisk = residual solvent. The descriptors "c" and "uc" in the parentheses denote signals arising from protons that are complexed and uncomplexed, respectively. After complexation with DB24C8, $H_h$ and $H_i$ of **5**-H·$PF_6$ are shifted by +0.90 and +0.34 ppm, respectively, indicating DB24C8 is flexible enough to complex $H_h$ and experiences no obvious hindrance from anthracene of **5**-H·$PF_6$.
